# Topic “Signaling Pathways in Liver Disease”

**DOI:** 10.3390/cells14020077

**Published:** 2025-01-08

**Authors:** Ralf Weiskirchen

**Affiliations:** Institute of Molecular Pathobiochemistry, Experimental Gene Therapy and Clinical Chemistry (IFMPEGKC), University Hospital Aachen, D-52074 Aachen, Germany; rweiskirchen@ukaachen.de

## 1. Introduction

Liver diseases pose a significant global health challenge, affecting millions of individuals and resulting in substantial morbidity and mortality. Conditions such as hepatitis, cirrhosis, fatty liver disease, and hepatocellular carcinoma (HCC) are among the most common liver conditions, often leading to serious complications like liver failure, portal hypertension, and increased susceptibility to infections. According to the World Health Organization (WHO), liver diseases contribute to approximately 2 million deaths annually worldwide, highlighting the urgent need for improved diagnostic tools and therapeutic strategies [1].

The complex nature of liver diseases is evident in their intricate pathogenesis, involving critical signaling pathways that regulate cellular responses to injury, inflammation, and fibrosis. These pathways are influenced by factors such as genetic predisposition, environmental exposures (e.g., alcohol consumption and viral infections), metabolic disturbances (such as obesity and diabetes), and immune system dysregulation. The interplay of these factors can lead to a series of events ultimately resulting in hepatic injury and disease progression [2].

Recent advancements in molecular biology have unveiled the intricate networks of signaling cascades pivotal in the development and progression of liver diseases. Key pathways like the mitogen-activated protein kinase (MAPK) pathway, nuclear factor kappa B (NF-κB) signaling, transforming growth factor-beta (TGF-β) signaling, and various apoptotic pathways have been identified as essential players in mediating hepatic inflammation, fibrosis, and tumorigenesis [3]. Understanding these signaling pathways not only elucidates the underlying mechanisms driving liver pathology but also presents opportunities for novel therapeutic interventions to mitigate disease progression.

In addition to traditional pharmacological approaches, emerging therapies targeting specific signaling molecules or pathways show promise for more effective treatment options. For example, the inhibitors of TGF-β signaling are under investigation for their potential to reduce fibrosis in chronic liver diseases (CLDs). Recent research indicates that the circadian clock plays a crucial role in regulating TGF-β signaling and liver fibrosis, with disruptions in circadian clock regulation leading to increased hepatic fibrosis [4]. Similarly, modulating immune responses through checkpoint inhibitors has shown efficacy in certain cases of HCC. These innovative strategies underscore the importance of ongoing research into the molecular foundations of liver disease [5].

**Figure 1:**
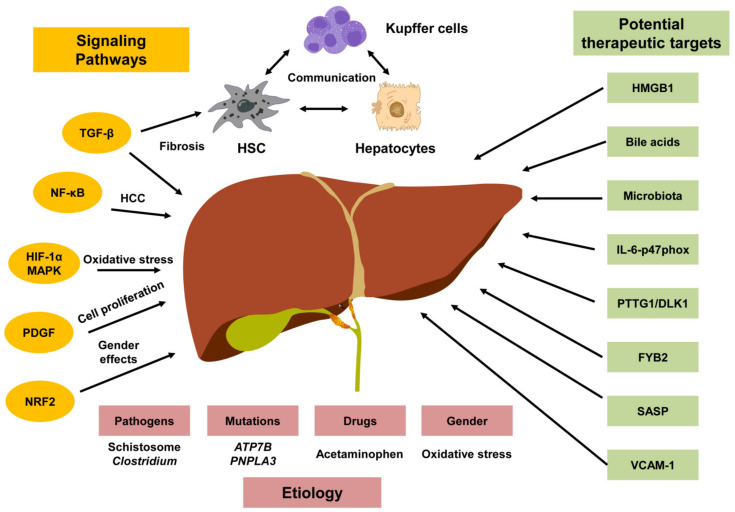
Signaling pathways and therapeutic insights in liver disease. The signaling pathways and key factors involved in liver diseases are presented in the Topic “Signaling Pathways in Liver Disease”. Major signaling pathways such as TGF-β, NF-κB, MAPK, PDGF, and NRF2 surrounding the liver are depicted, connecting to specific liver conditions including fibrosis and hepatocellular carcinoma (HCC). In these processes, different cell types, including hepatic stellate cells (HSCs), hepatocytes, and Kupffer cells communicate with each other. Potential etiologies and therapeutic targets emerging from the studies included in this Topic are indicated.

In this Topic titled “Signaling Pathways in Liver Disease”, 110 authors from eight countries (USA, UK, Germany, France, Italy, Spain, Greece, and China) provide a comprehensive overview of the recent research findings emphasizing the crucial role of signaling mechanisms in liver pathology (Figure 1).

This Topic presents a comprehensive collection of research that delves into the mechanisms of liver disorders. It features four insightful review articles that synthesize the current knowledge on various signaling pathways involved in liver disease, highlighting their roles in hepatocyte function, inflammation, and fibrosis. Each article explores various aspects of these complex signaling networks, from basic research elucidating fundamental biological processes to establishing novel clinically relevant targets. Additionally, this Topic aims to encourage further research efforts that will enhance our understanding of liver diseases and ultimately contribute to the development of more effective management strategies for affected individuals. My co-editors, Ruchi Bansal (University of Twente, The Netherlands), Gabriele Grassi (University of Trieste, Italy), and Leo A. van Grunsven (Vrije Universiteit Brussel, Belgium), and I hope that this compilation will serve as a valuable resource for researchers and clinicians dedicated to unraveling the complexities surrounding liver disease pathogenesis.

## 2. An Overview of Published Articles

The review by Zhong et al. (contribution 1) titled “From Inflammation to Fibrosis: Novel Insights into the Roles of High Mobility Group Protein Box 1 in Schistosome-Induced Liver Damage” discusses the significant role of High Mobility Group Box 1 (HMGB1) in schistosomiasis, a chronic parasitic disease that can lead to severe liver damage. The authors highlight that schistosome eggs trapped in the liver trigger granuloma formation, chronic inflammation, and eventual fibrosis, contributing to high morbidity and mortality rates. While praziquantel is effective against mature worms, there are limited options for reversing liver damage. HMGB1 is identified as a multifunctional cytokine involved in liver injury and immune responses by interacting with various receptors. Elevated levels of HMGB1 have been observed in patients with schistosomiasis, correlating with hepatic fibrosis development. The review emphasizes HMGB1’s potential as a therapeutic target due to its involvement in inflammatory pathways and fibrogenesis mediated by activated hepatic stellate cells (HSCs). The authors summarize the existing literature on HMGB1’s structure, functions, and mechanistic roles in both inflammation and fibrosis within the context of schistosomiasis. They suggest that inhibiting HMGB1 could provide protective effects against liver damage caused by schistosomes. Overall, the review calls for further research into HMGB1 as a potential treatment avenue for managing schistosome-induced liver diseases.

The article titled “Modulation of the Bile Acid Enterohepatic Cycle by Intestinal Microbiota Alleviates Alcohol Liver Disease” by Ciocan and colleagues (contribution 2) investigates the effects of pectin, a soluble fiber, on alcohol-induced liver disease (ALD) in mice. The study demonstrates that pectin supplementation improves liver health by reshaping the intestinal microbiota and enhancing bile acid (BA) metabolism. Pectin treatment led to decreased BA levels in plasma and liver while increasing their levels in the cecum, indicating enhanced BA excretion. This shift was associated with a change in BA composition towards less toxic hydrophilic forms. The beneficial effects of pectin were linked to an increase in intestinal bacteria with bile acid-metabolizing enzymes, which facilitated these metabolic changes. Furthermore, pectin influenced signaling pathways related to bile acids by inhibiting farnesoid X receptor (FXR) signaling in the ileum and impacting the expression of various BA transporters. Despite increased BA synthesis due to altered signaling, pectin promoted their excretion into feces. Overall, the findings suggest that dietary fiber like pectin could serve as a therapeutic strategy for managing ALD by modifying the gut microbiota and enhancing bile acid clearance. The authors advocate for further clinical trials to explore the potential of dietary interventions in ALD management among patients who often consume low-fiber diets.

In the article titled “Exercise Affects the Formation and Recovery of Alcoholic Liver Disease through the IL-6-p47^phox^ Oxidative-Stress Axis”, the team of Cui et al. (contribution 3) explores the impact of exercise on alcoholic liver disease (ALD) and its association with the interleukin-6 (IL-6)–p47^phox^ oxidative stress pathway. The study involved two experiments using male C57BL/6J mice, where ALD was induced through a high-fat alcoholic diet. In the first experiment, exercise intervention over six weeks following ALD model establishment showed that exercise significantly reduced serum triglycerides, improved liver function, and decreased inflammation in liver tissue. The combination of exercise with a NADPH oxidase inhibitor (apocynin) further enhanced these effects by reducing oxidative stress markers. In the second experiment, simultaneous alcohol consumption during exercise exacerbated dyslipidemia and oxidative stress, leading to increased liver injury. The findings highlighted that while exercise generally ameliorates ALD symptoms via the IL-6–p47^phox^ axis, concurrent alcohol intake during exercise negatively impacts lipid metabolism and increases oxidative stress levels. Overall, the review concludes that regular aerobic exercise can help mitigate hepatocyte damage and dyslipidemia in ALD through the modulation of inflammatory pathways, but drinking alcohol while exercising can counteract these benefits.

In the review article titled “Pituitary Tumor-Transforming Gene 1/Delta like Non-Canonical Notch Ligand 1 Signaling in Chronic Liver Diseases”, Perramón and Jiménez discuss the potential of targeting the pituitary tumor transforming gene 1/delta like non-canonical Notch ligand (PTTG1/DLK1) signaling axis as a therapeutic strategy for CLDs, including non-alcoholic fatty liver disease (NAFLD), liver fibrosis, and HCC (contribution 4). PTTG1 is identified as a proto-oncogene associated with cellular proliferation, inflammation, and fibrogenesis. DLK1, a target of PTTG1, has been shown to contribute to hepatic fibrosis by promoting the activation of HSCs. The authors highlight that both proteins are involved in regulating key processes such as metabolism, cell differentiation, and response to injury within the liver. The review discusses how the dysregulation of the PTTG1/DLK1 pathway is implicated in the progression of CLDs and suggests that inhibiting this signaling could mitigate the tissue remodeling and fibrosis associated with these conditions. The authors conclude that further research into this axis may provide new insights for developing effective treatments for CLDs.

The article “Caveolin-1 Alleviates Acetaminophen-Induced Hepatotoxicity in Alcoholic Fatty Liver Disease by Regulating the Ang II/EGFR/ERK Axis” explores the protective role of Caveolin-1 (CAV1) against liver injury caused by acetaminophen (APAP) in a model of alcoholic fatty liver disease (AFLD) (contribution 5). In their study, Xin and colleagues demonstrate that APAP exacerbates lipid accumulation and oxidative stress in AFLD, leading to increased levels of angiotensin II (Ang II) and decreased expression of CAV1 and ACE2. Through both in vivo and in vitro experiments, the researchers found that the overexpression of CAV1 alleviated APAP-induced hepatotoxicity by reducing Ang II levels and inhibiting the activation of the epidermal growth factor receptor (EGFR) and its downstream extracellular signal-regulated kinase (ERK) signaling pathway. Additionally, CAV1 was shown to restore autophagic flux, which is crucial for mitigating lipid accumulation. The findings suggest that targeting the CAV1-mediated regulation of the Ang II/EGFR/ERK axis could provide new therapeutic strategies for managing APAP-induced liver injury in patients with AFLD. Overall, this study highlights the potential of CAV1 as a protective factor against hepatic damage associated with alcohol consumption and APAP overdose.

The article titled “Role of Hepatocyte Senescence in the Activation of Hepatic Stellate Cells and Liver Fibrosis Progression” explores the relationship between hepatocyte senescence and liver fibrosis, with a particular focus on how senescent hepatocytes influence HSC activation (contribution 6). Wijaysiri and colleagues conducted an analysis of liver biopsy specimens from patients with NAFLD and found a significant correlation between the presence of senescent hepatocytes (marked by p16 expression) and HSC activation (indicated by αSMA expression) as well as the fibrosis stage. Using in vitro models, the researchers discovered that conditioned media from senescent HepG2 cells significantly upregulated inflammatory and fibrogenic gene expression in cultured HSCs, suggesting that the factors secreted by senescent hepatocytes activate these cells. Notably, the platelet-derived growth factor (PDGF) levels were higher in media from the senescent cells compared to controls. The findings support a causal link between hepatocyte senescence and liver fibrosis progression through the secretion of senescence-associated secretory phenotype (SASP) factors. The authors propose that targeting this pathway could offer new therapeutic strategies for managing CLDs, emphasizing the importance of understanding cellular mechanisms in liver pathology.

The article by Charbonnier titled “*ATP7B*-Deficient Hepatocytes Reveal the Importance of Protein Misfolding Induced at Low Copper Concentration” examines the role of *ATP7B*, a copper transporter, in hepatocyte function and its implications for Wilson disease (contribution 7). The study employs CRISPR/Cas9 technology to generate *ATP7B*-deficient HepG2/C3a cell lines to explore how these cells respond to copper exposure compared to their wild-type counterparts. Key findings indicate that *ATP7B* deficiency leads to increased sensitivity to copper-induced stress, resulting in significant protein misfolding and the enhanced expression of heat shock proteins like HSPA6. The research highlights that low concentrations of copper trigger oxidative stress responses and activate critical signaling pathways involving Ang II, EGFR, and ERK1/2, which are linked to liver injury and fibrosis progression. Furthermore, the study shows that CAV1 can mitigate APAP-induced lipid accumulation in AFLD by regulating these signaling pathways. Overall, the findings suggest that targeting the mechanisms related to *ATP7B* may provide new therapeutic strategies for managing copper-related liver diseases such as Wilson’s disease.

In the article “FYB2 Is a Potential Prognostic Biomarker for Hepatocellular Carcinoma”, authored by Qu and colleagues, the role of FYN-binding protein 2 (FYB2, C1orf168) as a prognostic biomarker in HCC is analyzed (contribution 8). The study reveals that FYB2 expression is significantly downregulated in HCC tissues compared to normal liver tissues. Lower levels of FYB2 correlate with poorer survival outcomes, advanced tumor grades, and higher pathological stages. Using bioinformatics analyses from public databases such as TCGA and GEO, the researchers found that FYB2 can serve as an independent prognostic factor alongside AJCC-M staging. Gene Set Enrichment Analysis (GSEA) indicated that FYB2 is associated with cellular metabolism-related pathways and cancer regulation. Single-cell transcriptome analysis showed that FYB2-positive cells are primarily located in hepatocytes. Spatial transcriptomics revealed higher FYB2 expression in adjacent non-tumor areas compared to tumor regions. The findings suggest that targeting the mechanisms involving FYB2 could provide new therapeutic strategies for HCC. However, further validation in larger clinical cohorts and the exploration of the biological mechanisms behind FYB2’s role in tumorigenesis are necessary. Overall, this study highlights the potential of FYB2 as a valuable biomarker for prognosis and treatment decision making in patients with HCC.

The contribution “Oxygen Gradient Induced in Microfluidic Chips Can Be Used as a Model for Liver Zonation” by Ghafoory et al. presents a novel microfluidic system designed to create oxygen gradients that mimic the conditions found in liver acini (contribution 9). The study aims to explore how these gradients affect hepatocyte function, specifically focusing on the expression of hypoxia-inducible factor 1-alpha (Hif1α) and albumin. Utilizing interconnected microfluidic chips, the authors established a controlled flow of media that allowed for the measurement of oxygen levels over time. They observed a significant reduction in oxygen concentration from inlet to outlet, which correlated with increased Hif1α expression and decreased albumin production in HepG2 cells, indicating that even slight changes in oxygen levels can trigger metabolic responses relevant to liver zonation. The study also demonstrated that conditioned media from senescent HepG2 cells could activate HSCs, suggesting a link between hepatocyte senescence and liver fibrosis progression. Overall, this research provides insights into the cellular mechanisms underlying liver zonation and offers a promising platform for studying liver metabolism and pathology in vitro, paving the way for future investigations into therapeutic strategies for liver diseases.

The article “The Clostridium Metabolite P-Cresol Sulfate Relieves Inflammation of Primary Biliary Cholangitis by Regulating Kupffer Cells” by Fu et al. evaluates the role of p-Cresol sulfate (PCS), a metabolite produced by *Clostridium*, in alleviating inflammation associated with primary biliary cholangitis (PBC) (contribution 10). The study highlights that PCS levels are significantly reduced in PBC patients and animal models, particularly at advanced stages of the disease. Using both in vivo and in vitro experiments, the researchers demonstrated that dietary supplementation with tyrosine, which increases PCS levels, led to decreased liver inflammation and improved inflammatory cytokine profiles in PBC mice. The findings indicate that PCS modulates the polarization of hepatic macrophages (Kupffer cells), shifting them from a pro-inflammatory M1 phenotype to an anti-inflammatory M2 phenotype. The study suggests that PCS could serve as a potential therapeutic agent for managing PBC by regulating immune responses within the liver. Additionally, decreased levels of PCS might serve as an early diagnostic marker for PBC onset. Overall, this research underscores the importance of microbial metabolites like PCS in liver health and disease management.

The article titled “PNPLA3(I148M) Inhibits Lipolysis by Perilipin-5-Dependent Competition with ATGL” by Witzel et al. investigates the role of the Patatin-Like Phospholipase Domain Containing Protein 3 (*PNPLA3*) I148M polymorphism in lipid metabolism and its implications for liver diseases, particularly steatohepatitis (contribution 11). The study reveals that the I148M variant of *PNPLA3* negatively affects lipolysis by competing with adipose triglyceride lipase (ATGL) for binding to perilipin-5, a protein crucial for lipid droplet (LD) metabolism. Using a combination of human liver biopsies, mouse models, and cell culture experiments, the researchers demonstrated that hepatocytes carrying the I148M variant exhibited increased lipid accumulation and impaired lipolytic activity. Immunohistochemical analyses showed that PNPLA3 localized to LDs in patients with steatosis and inflammation, correlating with disease severity. The findings suggest that the interaction between PNPLA3, perilipin-5, and ATGL is critical in regulating lipid metabolism in the liver. The authors propose that targeting this pathway may provide therapeutic opportunities for managing fatty liver diseases associated with the *PNPLA3* polymorphism. Overall, this study enhances our understanding of how genetic variations impact lipid metabolism and the progression of liver disease.

In their article titled “Cellular Senescence in Hepatocellular Carcinoma: The Passenger or the Driver?”, the Cai research team explores the complex role of cellular senescence in the progression of HCC (contribution 12). The authors discuss how senescence, characterized by stable cell cycle arrest, can have dual effects on liver health, acting as a protective mechanism against tumorigenesis while also contributing to inflammation and fibrosis that promote cancer development. The review highlights that senescent hepatocytes secrete a variety of factors known as SASP, which can influence neighboring cells and drive fibrogenesis. It emphasizes the importance of understanding how senescent cells interact with immune cells and other components of the liver microenvironment during HCC progression. Additionally, the article discusses potential therapeutic strategies targeting cellular senescence, including inducing or eliminating senescent cells and modulating SASP factors. The authors argue for further investigation into specific biomarkers for senescence and its role in drug resistance to improve treatment outcomes for HCC patients. Overall, this review underscores that cellular senescence is not merely a consequence but may actively drive liver carcinogenesis, suggesting new avenues for diagnosis and therapy in HCC management.

The article titled “Analysis of the Role of Stellate Cell VCAM-1 in NASH Models in Mice” by Chung et al. analyzes the role of vascular cell adhesion molecule-1 (VCAM-1) expressed in HSCs during the development and progression of non-alcoholic steatohepatitis (NASH) (contribution 13). The researchers utilized two different mouse models to explore whether VCAM-1 influences liver inflammation, fibrosis, and steatosis. The study found that while VCAM-1 expression was upregulated in HSCs during NASH, HSC-specific deletion of VCAM-1 did not significantly affect steatosis, inflammation, or fibrosis in either model used. This suggests that VCAM-1 on HSCs is dispensable for NASH development. However, the authors noted that other adhesion molecules might compensate for the absence of VCAM-1. Overall, the findings indicate that although VCAM-1 is associated with activated HSCs during NASH, it does not play a critical role in mediating liver damage or fibrogenesis in the context of the two experimental models studied. The research highlights the need for further exploration into other potential mechanisms and factors involved in NASH pathology.

Finally, the article titled “Sex Differences Affect the NRF2 Signaling Pathway in the Early Phase of Liver Steatosis: A High-Fat-Diet-Fed Rat Model Supplemented with Liquid Fructose” by Di Veroli et al. evaluates how sex differences influence the Kelch-like ECH-associated protein 1/Nuclear factor erythroid 2-related factor 2 (KEAP1/NRF2) signaling pathway in liver steatosis induced by a high-fat diet and liquid fructose (contribution 14). The study utilized male and female Sprague Dawley rats, feeding them either a control diet or a high-fat–high-fructose diet for three months. Key findings indicate that female rats exhibited significant hepatic steatosis characterized by increased lipid levels, while males showed a more resilient metabolic phenotype despite similar dietary intake. The researchers found that NRF2 expression was upregulated in males but downregulated in females under dietary conditions, suggesting that males have a better capacity to cope with oxidative stress through enhanced autophagy and antioxidant defenses. Furthermore, the study demonstrated that while both sexes activated autophagic processes, only males displayed proper autophagic flux. In contrast, females showed impaired responses to endoplasmic reticulum stress markers and reduced activity of antioxidant proteins such as NQO1 and HO-1. Overall, this research highlights the importance of considering sex differences when studying metabolic disorders like metabolic dysfunction-associated steatotic liver disease (MASLD). The findings suggest potential pathways for developing targeted therapies that take into account these differences to improve treatment outcomes for MASLD patients.

## 3. Concluding Remarks

The collection of articles reviewed presents a comprehensive exploration of various mechanisms and factors involved in liver diseases, with a particular focus on conditions such as NAFLD/MASLD, ALD, HCC, and PBC. Key themes emerge across the studies. Several articles delve into specific molecular pathways, including the KEAP1/NRF2 axis, which plays a crucial role in antioxidant defense and cellular responses to oxidative stress. The dysregulation of this pathway is linked to increased susceptibility to liver damage and fibrosis. The role of cellular senescence is highlighted as both a protective mechanism against tumorigenesis and a contributor to chronic inflammation and fibrosis in the liver. The SASP can influence neighboring cells, promoting either tissue repair or exacerbating disease progression. Genetic polymorphisms, such as PNPLA3 (I148M), are shown to significantly impact lipid metabolism and the progression of fatty liver diseases. These genetic variations can lead to altered interactions between key proteins involved in lipolysis, contributing to steatosis and inflammation. Additionally, several studies emphasize the importance of sex differences in metabolic responses and disease progression, indicating that males and females may exhibit distinct biochemical pathways when exposed to similar dietary challenges or stressors. The potential for targeting specific pathways, such as inhibiting pro-inflammatory cytokines or modulating autophagy, emerges as a promising strategy for developing effective treatments for CLDs. Furthermore, dietary interventions aimed at regulating metabolite levels show potential for therapeutic benefits.

In conclusion, these articles collectively underscore the complexity of liver pathophysiology where multiple factors, including genetics, cellular behavior, environmental influences, and sex-specific responses, interact to drive disease progression. A deeper understanding of these mechanisms not only enhances our knowledge of liver diseases but also paves the way for innovative therapeutic strategies tailored to individual patient profiles based on their unique biological contexts. Future research should continue exploring these intricate relationships with an aim toward personalized medicine approaches in managing liver-related disorders.

It should be noted that this Topic attracted a significant number of submissions, indicating that liver fibrosis remains a prominent subject in Hepatology research. As a result, the publisher has tasked me with editing a second Topic focused on this field. The new Topic titled “Signaling Pathways in Liver Disease 2nd Edition” is now accepting submissions. I warmly invite research articles, reviews, and concise perspective pieces from experts covering all facets related to this topic.

## Data Availability

No new data were created or analyzed in this study. Data sharing is not applicable to this article.

## Abbreviations

The following abbreviations are used in this manuscript:

AFLD	Alcoholic fatty liver disease
ALD	Alcohol-induced liver disease
Ang II	Angiotensin II
APAP	Acetaminophen
ATGL	Adipose triglyceride lipase
BA	Bile acid(s)
CAV1	Caveolin-1
CLD(s)	Chronic liver disease(s)
DLK1	Delta like non-canonical Notch Ligand
EGFR	Epidermal growth factor receptor
ERK	Extracellular-signal-regulated kinase
FYB2	FYN-binding protein 2
HCC	Hepatocellular carcinoma
Hif1α	Hypoxia-inducible factor 1-alpha
HMGB1	High Mobility Group Box 1
HSC(s)	Hepatic stellate cell(s)
IL-6	Interleukin 6
KEAP1/NRF2	Kelch-like ECH-associated protein 1/Nuclear factor erythroid 2-related factor 2
LD(s)	Lipid droplet(s)
MAPK	Mitogen-activated protein kinase
MASLD	Metabolic dysfunction-associated steatotic liver disease
NAFLD	Non-alcoholic fatty liver disease
NASH	Non-alcoholic steatohepatitis
PBC	Primary biliary cholangitis
PCS	p-Cresol sulfate
PNPLA3	Patatin-Like Phospholipase Domain Containing Protein 3
PTTG1	Pituitary tumor transforming gene 1
SASP	Senescence-associated secretory phenotype
TGF-β	Transforming growth factor-beta
VCAM-1	Vascular cell adhesion molecule-1
